# The influence of bracket torque on external apical root resorption in bimaxillary protrusion patients: a retrospective study

**DOI:** 10.1186/s12903-022-02042-3

**Published:** 2022-01-11

**Authors:** Xiaojuan Zhang, Hong Zhou, Xiangling Liao, Yi Liu

**Affiliations:** 1 Department of Oral, Beijing Luhe Hospital, Capital Medical University, 82 Xinhua South Road, Beijing, 101100 People’s Republic of China; 2 Department of Radiology, Beijing Luhe Hospital, Capital Medical University, Beijing, 101100 People’s Republic of China; 3 Laboratory of Tissue Regeneration and Immunology and Department of Periodontics, Beijing Key Laboratory of Tooth Regeneration and Function Reconstruction, School of Stomatology, Capital Medical University, Tian Tan Xi Li No.4, Beijing, 100050 People’s Republic of China

**Keywords:** Self-ligating brackets, Torque, Root resorption

## Abstract

**Background:**

To evaluate the difference in root resorption between standard torque self-ligating brackets and high torque self-ligating brackets in bimaxillary protrusion patients after orthodontic treatment.

**Methods:**

Pre-treatment and post-treatment Cone beam computed tomography (CBCT) of 32 patients (16 treated with the high torque DamonQ 0.022″ bracket and 16 with the 0.022″ standard torque self-ligating bracket) were selected. The first premolars were extracted from all patients before treatment. After mini-screw implants were inserted into the buccal region between the second premolar and first molar, 150 g of force was applied to retract the upper and lower anterior teeth to close the extraction space on each side. CBCT images of all patients were taken before and after treatment. Three-dimensional reconstruction of the maxillary central incisor, lateral incisor and canine was conducted with Mimics 20.0 software. The volumes of the roots were calculated using Gomagics Studio 12.0 software. The differences between the pre-treatment and post-treatment root volumes were statistically evaluated with a paired-samples t-test.

**Results:**

There was no statistically significant difference in root resorption degree between the two kinds of torque brackets. The patient’s degree of root resorption in the high torque self-ligating group was greater than that in the standard torque group.

**Conclusions:**

There was no significant difference in root external apical resorption between the high torque self-ligating brackets and the standard torque self-ligating brackets in bimaxillary protrusion patients.

## Background

Root resorption is one of the frequent complications of orthodontics. The prevalence of root resorption varies from 20 to 100% among orthodontic patients [1]. It has also been of great concern to orthodontic doctors in recent years. Orthodontic root resorption involves external apical root resorption (EARR) (common in the tip of the root), which is the pathological process associated with cementum and dentin loss. The factors affecting root resorption include genetics, ethnicity, sex, age, the type of malocclusion, treatment time, the type of brackets, the direction and the magnitude of force, premolar extractions or not, and amount of apical displacement, which are all risk factors for EARR [2–6]. There has been a large amount of research on root resorption in recent years. In terms of appliances, the impact of root resorption has been reported, such as edgewise appliances, Begg techniques, and invisible appliances [7–10]. The effect of self-ligating brackets and conventional brackets on root resorption has been reported [11–13]. However, there are no prior reports on the effect of torque self-ligating brackets on root resorption. A number of previous studies on root resorption was performed using conventional radiographs, such as periapical and panoramic radiographs. These images are two-dimensional. However, root resorption changes in three-dimensional. The Two-dimensional (2D) images cannot detect root resorption on every surface. In addition, due to the magnification errors, geometric distortion and superposition they were not accurate to evaluate the amount of resorptionhave. Therefore the 2D images have some limitations in the accuracy of EARR measurement. CBCT is more gigantic advantages in the accuracy for odiagnosis and measurement of root resorption [14]. As an effective imaging method, CBCT is used to diagnose orthodontic root resorption. It is increasingly being used in the study of root resorption. CBCT was used to three-dimensional reconstruction with no the structural superimposition [15]. We can observe the images at all angles by using Three-dimensional (3D) reconstruction. Root resorption actually occurs in three dimensions, including on the buccal-lingual and mesial-distal sides, and the degree of absorption of each surface is different. Therefore, the volume index is more accurate than the length index in reflecting the degree of root resorption. Wang [16] has proved this point.

In view of the above, the aim of this study was to explore the different effects of root resorption in patients with maxillary protrusion using different torque brackets. Furthermore, we analysed the effects of the distance of root tip movement and the treatment time on root resorption.

## Methods

This study was designed as a retrospective study, and it was conducted according to the Declaration of Helsinki principles. The study protocol was approved by the Ethics Committee of Beijing Luhe Hospital, Capital Medical University (2018LH-KS-008). Written informed consent was obtained from each participants before participating in the study.

Based on a retrospective power analysis, a total of 28 patients were required to demonstrate a clinically meaningful difference in root resorption between the appliance systems, with a 0.05 significance level and a power of 80%.

In this retrospective study, 214 participants were screened in the Department of Orthodontics, Beijing Luhe Hospital, Capital Medical University. A total of 172 participants were excluded because they did not meet the inclusion criteria. Finally, 32 patients were enrolled in this study. They were divided into two groups according to the different torque brackets: a high torque self-ligating bracket group with 16 patients (Damon 3, ORMCO, USA) and a standard torque self-ligating bracket group with 16 patients. For the bracket torque data, see Table 1.

**Table 1:** The bracket torque data of the two groups

	Maxillary central incisor	Maxillary lateral incisor	Maxillary canine
High torque brackets	11°	13°	22°
Standard torque brackets	7°	6°	15°

## Inclusion and exclusion criteria

### Inclusion criteria

1. Patients aged 18–30 years
2. Light or moderate anterior crowding with bimaxillary protrusion
3. CBCT was performed before and after treatment
4. All teeth erupted pre-treatment, the teeth were healthy, the maxillary incisors were without pulp disease and periapical disease, and there was no obvious root resorption
5. Extracted the four first premolars and implanted mini-screw implants

### Exclusion criteria

1. Severe anterior crowding
2. Impacted teeth
3. Treatment of patients with anterior tooth trauma
4. Patients with hypoplasia
5. Patients who required orthognathic surgery or had already had surgery
6. Treatment with a conventional edgewise appliance
7. Did not receive extraction treatment

## Treatment procedure

All 32 patients were treated with fixed orthodontic treatment, and the 4 first premolars were removed before treatment. The archwire sequence was 0.014-in, 0.014 × 0.025-in, 0.019 × 0.025-in copper-nickel-titanium Damon (Ormco) and finished with 0.019 × 0.025-in stainless steel. The first and second premolars of the upper and lower jaws were implanted with planting nails. Mini-screws (Ningbo Cibei Medical Treatment Appliance Co., Ltd., China; diameter: 1.6 mm; implant length: 11 mm; screw length: 7 mm) were placed between the second premolar and the first molar buccal to close the extraction space. The treatment completion time ranged from 20 to 32 months, with an average of 27.9 months. All patients were examined with the same CBCT machine (Planmeca Romexis, Finland; 0.2-mm voxel size, 90 kV, 8.0 mA, 13.755 s exposure time, and 351 × 351 × 410 exposure field) and were operated on by the same dentist.

## Method of measurement

**Fig. 1:**
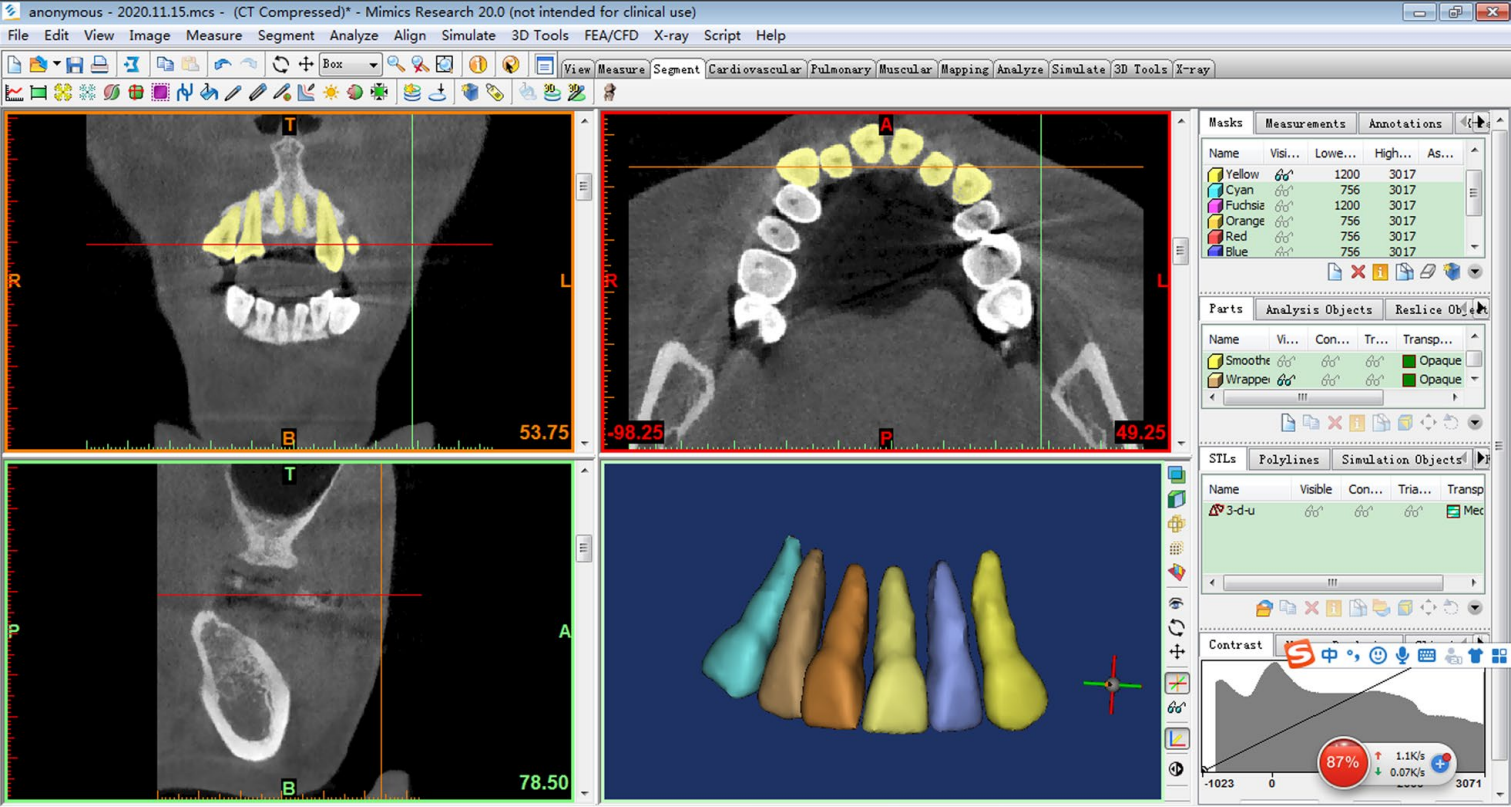
Three-dimensional reconstruction

**Fig. 2:**
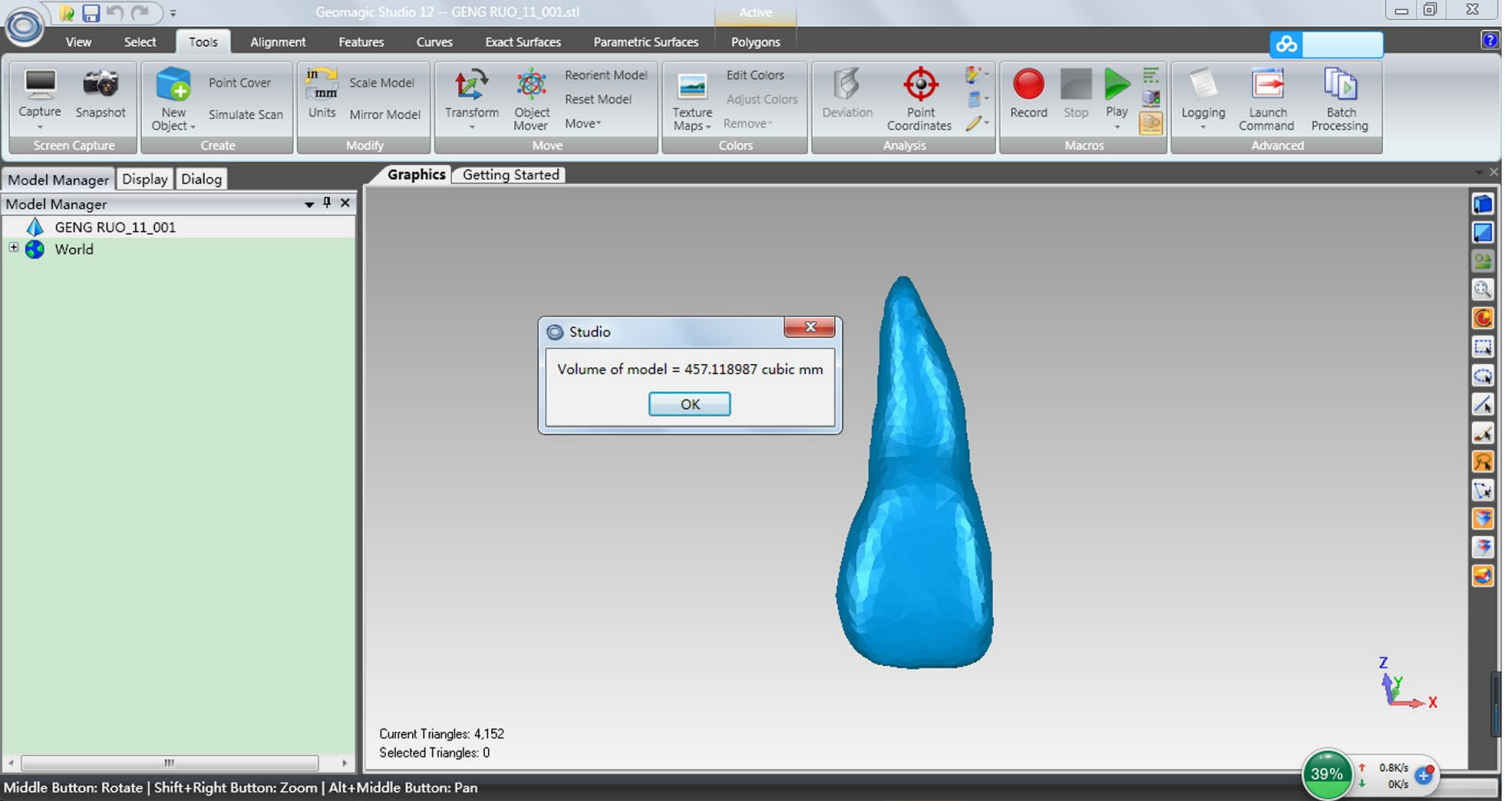
Volume calculation

Measurement of tooth volume: the CBCT data of the patients were imported into Mimics 20.0 software (Materialise, Leuven, Belgium), selecting the appropriate threshold for a single tooth reconstruction. The reconstructed teeth were exported to an STL file. Then, the STL file was imported into Gomagics Studio 12.0 (Materialise) for volume calculations (Figs. 1, 2).

### Error study

The measurements were performed by the same imaging specialist. After 20 days of measurement, 10 randomly selected images were repeated for three-dimensional reconstruction and measurement. The measurement error was calculated by intraclass correlation coefficient (ICC) statistics. We calculated the intra-examiner consistency. According to Roberts and Richmond [17], the reliability is excellent if the ICC value is higher than 0.75, acceptable if it is between 0.4 and 0.75, and low if the ICC is smaller than 0.4. In this study, the ICC showed excellent intra-examiner reliability. The ICC for volume measurements showed an average of 95.7%, with a range from 0.875 to 0.984, and the method presented high reproducibility.

### Statistical analyses

A paired t-test was conducted to compare the degree of root resorption in each group before and after treatment. A unpaired t-test was used to assess the differences between groups. All of the data are expressed as the means with standard deviations, and the significance level was set at 5%. Statistical calculations were performed with SPSS 20.0 (IBM Inc., USA).

## Results

ICC statistics showed that there was almost perfect consistency between the two measures of root resorption upon the inspector evaluation. There were no statistically significant systematic errors. The casual errors were within the acceptable ranges.

There was no statistically significant difference in the comparison of the initial ages or the treatment time between the high torque group and the standard torque group (Table 2).

**Table 2:** Comparison of initial ages and treatment time between the high torque group and the standard torque group

Variable	High torque group		Standard torque group		*P*
	Mean	SD	Mean	SD	
Initial age (years)	24.23	4.76	24.71	5.46	0.486
Treatment time (months)	26.9	3.72	25.32	4.60	0.206

In the comparison between the two groups, there was a significant difference in all tooth volumes before and after treatment between the two groups (Tables 3 and 4). Root resorption occurred, and there was a significant difference after treatment in the two groups.

**Table 3:** The degree of root resorption (mm^3^) between pre-treatment and post-treatment for the patients with high torque

Measurements (mm^3^)	Mean	SD	Mean	SD	*P*
Maxillary right central incisor	479.47	36.51	454.98	38.02	0.000
Maxillary right lateral incisor	336.24	24.43	314.84	21.52	0.000
Maxillary right canine	521.59	52.84	498.64	50.99	0.000
Maxillary left central incisor	480.41	33.81	455.35	32.56	0.000
Maxillary left lateral incisor	341.75	24.34	320.21	20.83	0.000
Maxillary left canine	514.59	52.84	490.91	49.96	0.000

**Table 4:** The degree of root resorption (mm^3^) between pre-treatment and post-treatment for the patients in the standard torque group

Measurements (mm^3^)	Pre-treatment (T2)		Post-treatment (T2)		*P*
	Mean	SD	Mean	SD	
Maxillary right central incisor	482.47	36.52	460.81	34.28	0.000
Maxillary right lateral incisor	338.55	24.34	318.99	23.83	0.000
Maxillary right canine	517.09	52.84	497.36	48.39	0.000
Maxillary left central incisor	483.22	33.81	461.12	32.04	0.000
Maxillary left lateral incisor	339.18	24.12	319.98	23.54	0.000
Maxillary left canine	516.58	52.42	494.89	49.51	0.000

Although the root resorption of the high torque group was higher than that of the standard torque group, there was no statistically significant difference in the degree of root resorption (Table 5). After the treatment, the two groups of teeth had different root resorptions of the same teeth. However, there was no statistically significant difference.

**Table 5:** The difference in root resorption between the high torque and standard torque groups

Measurements (mm^3^)	High torque group (T1)		Standard torque group (T2)		*P*
	Mean	SD	Mean	SD	
Maxillary right central incisor	24.48	6.31	21.66	3.75	0.135
Maxillary right lateral incisor	21.41	4.1	19.57	2.41	0.133
Maxillary right canine	22.95	5.4	19.73	5.33	0.100
Maxillary left central incisor	25.07	4.52	22.09	4.54	0.073
Maxillary left lateral incisor	21.54	5.65	19.20	4.14	0.191
Maxillary left canine	23.48	5.69	21.69	6.11	0.398

## Discussion

Root resorption is one of the common complications of orthodontics, and it is also the focus of orthodontic clinical research. Previous studies have shown that the degree of root resorption is lower in patients treated with self-ligating brackets than in those treated with traditional brackets [18]. Many studies have shown that there is no difference in root resorption between self-ligating brackets and traditional brackets [19–21]. However, the self-ligating bracket has many advantages, such as a light force and a low friction force. They can reduce the operating time in the chair and bring comfort to the patients. They are widely used in orthodontics. With the development of self-ligating brackets, brackets with different torque angles have been introduced to meet the needs of clinical treatment. This experiment was designed to detect the effect of high torque brackets and standard torque brackets on root resorption in bimaxillary protrusion patients.

With the wide application of CBCT, an increasing number of studies have been performed to evaluate root resorption by CBCT [22–24]. CBCT can accurately measure tooth and root resorption volumes, and it is a more accurate and reliable 3D measuring method for root resorption [19]. CBCT measures root resorption more often than on X-rays [25]. At present, most of the research on root resorption relies on length measurements. However, root resorption is not just a two-dimensional variation in length. It includes changes in the three-dimensional direction of the buccal tongue and the proximal middle, and the absorption on each side is slightly different. Therefore, taking the volume of the tooth used to describe the amount of root absorption is more appropriate. It can more accurately reflect root resorption [26–29]. However, research in this area is still relatively limited. To better assess the degree of root resorption, this study also used the method of measuring the tooth volume to measure the degree of root resorption before and after treatment.

Patients with extracted teeth are more likely to develop root resorption than those who do not receive orthodontics [30–32]. In this study, all of the patients had the first premolar removed before treatment. The same orthodontist provided the same treatment, including a similar arch wire replacement throughout the entire treatment process. Mini-screw implants were inserted into the buccal region between the second premolar and first molar.

Excessive force is one of the factors involved in severe root resorption [33]. In this study, to avoid excessive force causing root resorption, 150 g of force was applied to retract the upper and lower anterior teeth to close the extraction space on each side [34]. All of the patients underwent CBCT by the same radiologist under the same parameters before and after treatment, which ensured good comparability between the two groups. In addition, we selected more patients (32,192 teeth) to reduce the study error caused by a small sample size.

In this study, we only considered the maxillary anterior teeth. On the one hand, the torque of the brackets in the two groups was different for the maxillary anterior teeth. On the other hand, the maxillary anterior teeth are the most prone to root resorption in orthodontic treatment [35–37]. Although the torque on the mandibular teeth was different from the standard torque, it was not included in this study. For patients with bimaxillary protrusion, a large number of anterior teeth were moved to improve the degree of protrusion [38]. Therefore, it is necessary to control the torque of the anterior teeth to acquire the desired tooth position [39]. The maxillary anterior teeth show the movement tendency of the coronal lip and the root tongue when using the high torque bracket, which is helpful to prevent torque loss in the anterior teeth. The stress expression value of the perimembrane in the high torque bracket was obviously higher than that of the standard torque bracket [40]. Case et al. [41] reported that the effect of torque force on root resorption showed that the greater the force, the greater the root resorption scope.

In this study, the average root absorption of the high torque bracket group and the standard torque bracket group were 23.15 mm^3^ and 20.34 mm^3^, respectively. Compared with the standard torque bracket, the root resorption of the high torque self-ligating bracket was slightly higher than that of the standard torque bracket. However, the difference between them was not statistically significant. In the relatively severe root resorption between the two groups, the number and quantity of moderate and severe root resorption in the high torque group were both greater than those in the standard torque group. Yangxue et al. [42] also showed that more torque control in the maxillary anterior teeth of the high torque group led to overall and partial root control and higher root resorption.

In this study, we found that there was no statistically significant difference in the treatment time between the groups. However, a longer treatment time led to more root resorption, which is consistent with previous studies [20, 43, 44]. Treatment duration is a risk factor for the development of severe EARR. However, some authors disagree [2, 45].

Some of the shortcomings of this study need attention. First, although we strictly matched the treatment group and the control group when choosing the cases, it may be difficult to avoid the effects of confounding factors on the results. It is best to compare the root resorption of the two types of brackets by random selection in the future. Second, although the detection process has increased the sample size compared with previous studies, the sample size is still insufficient. In future studies, we will continue to increase the sample size to correct for the effect of the sample size on the results. Finally, we still need to include more patients of different races to verify whether the two brackets affect root resorption.

## Conclusion

There was no significant difference in external apical root resorption between patients treated with the high torque Damon self-ligating bracket and the high torque Damon self-ligating bracket.

## Data Availability

The datasets used and/or analyzed during this study belongs to the authors and are available from the corresponding author only upon on reasonable request.

## Abbreviations

EARR: External apical root resorption
CBCT: Cone beam computed tomography
ICC: Intraclass correlation coefficient
2D: Two-dimensional
3D: Three-dimensional
